# Intestinal Lymphangiectasia Associated With Refractory Ascites in a Cirrhosis Patient

**DOI:** 10.7759/cureus.12567

**Published:** 2021-01-07

**Authors:** Ramesh Kumar, Tarun Kumar, Utpal Anand, Rajeev N Priyadarshi

**Affiliations:** 1 Gastroenterology, All India Institute of Medical Sciences Patna, Patna, IND; 2 Pathology/Lab Medicine, All India Institute of Medical Sciences Patna, Patna, IND; 3 Surgical Gastroenterology, All India Institute of Medical Sciences Patna, Patna, IND; 4 Radiology, All India Institute of Medical Sciences Patna, Patna, IND

**Keywords:** lymphangiectasia, refractory ascites, cirrhosis, portal hypertension

## Abstract

Lymphatic systems play a very important role in the body fluid homeostasis by interstitial fluid reabsorption. Lymphatic dysfunctions are common in patients with advanced cirrhosis, contributing to ascites and lymphedema. An unusual manifestation of lymphatic dysfunction in patients with cirrhosis is intestinal lymphangiectasia. A sustained rise in lymphatic pressure secondary to portal hypertension often contributes to the rupture of intestinal lymphangiectasia, resulting in the loss of plasma proteins, lymphocytes and lipids via the release of lymph into the intestinal lumen. Therefore, in addition to lymphatic pump failure, lymphangiectasia can lead to further worsening of ascites by causing severe hypoalbuminemia. On endoscopy, lymphangiectasia appears as whitish distended villi in the duodenum. Dietary changes, which include low-fat diet and medium-chain fat, are currently the cornerstone of lymphangiectasia therapy. We report here an interesting case of cirrhosis with recent worsening of ascites associated with severe intestinal lymphangiectasia and splenomesentric venous thrombosis.

## Introduction

The lymphatic vascular system is essential for tissue fluid homeostasis [1]. By returning excess tissue fluid back to the bloodstream, it keeps tissues clear of edema. The main contributor to intravascular colloid oncotic pressure (COP) is serum albumin that helps fluid to remain within the vascular compartment. Hypoalbuminemia can thus lead to the deterioration of ascites. In patients with cirrhosis, there is a disturbance in microcirculatory exchange of fluid and protein, and sustained portal hypertension (PHT) gradually overwhelms the safety factors of intravascular COP and the lymphatic compensatory mechanism leading to ascites and edema [2]. PHT not only induces a substantial increase in lymph output from the liver and intestines, but also an increase in lymph pressure secondary to PHT may lead to dilatation of intestinal lymphatic known as intestinal lymphangiectasia (IL) [3].

Intestinal lymph mainly contains proteins, lipoproteins and lymphocytes that are transported to the systemic circulation via lymphatic vessels. A prolonged rise in intestinal lymphatic pressure leads to rupture of dilated intestinal lymphatic and leakage of lymph into the lumen of the intestines, leading to hypoproteinemia, hypoalbuminemia, lymphocytopenia and hypogammagloblinemia [4,5]. Hence, in patients with advanced cirrhosis, IL can lead to worsening of ascites by causing severe hypoalbuminemia, in addition to the increase lymph flow and decrease lymphatic return. Serum albumin is also required for the proper functioning of furosemide, a widely used diuretic [6]. In addition, disruption of lymphatic flow in IL leads to malabsorption of long-chain fats and fat-soluble vitamins, and loss of lymphocytes contributes to increased susceptibility to infection [7]. It is not clear why IL does not develop in all patients with cirrhosis, despite sustained PHT. There might be some extra abnormalities required for that. We report here a case of cirrhosis with recent worsening of ascites associated with severe IL and splenomesentric venous thrombosis.

## Case presentation

**Figure 1:**
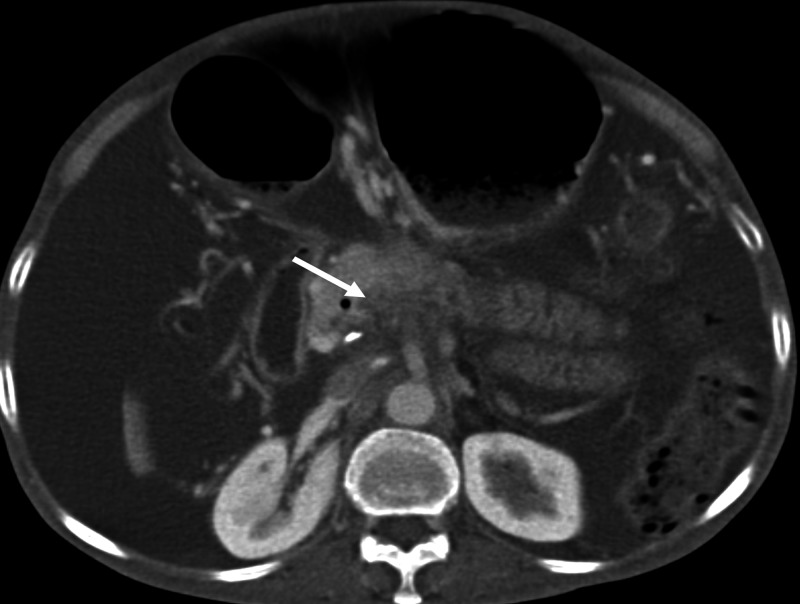
A computed tomography scan of the abdomen demonstrating thrombosed splenic and superior mesenteric veins with collaterals around.

**Figure 2:**
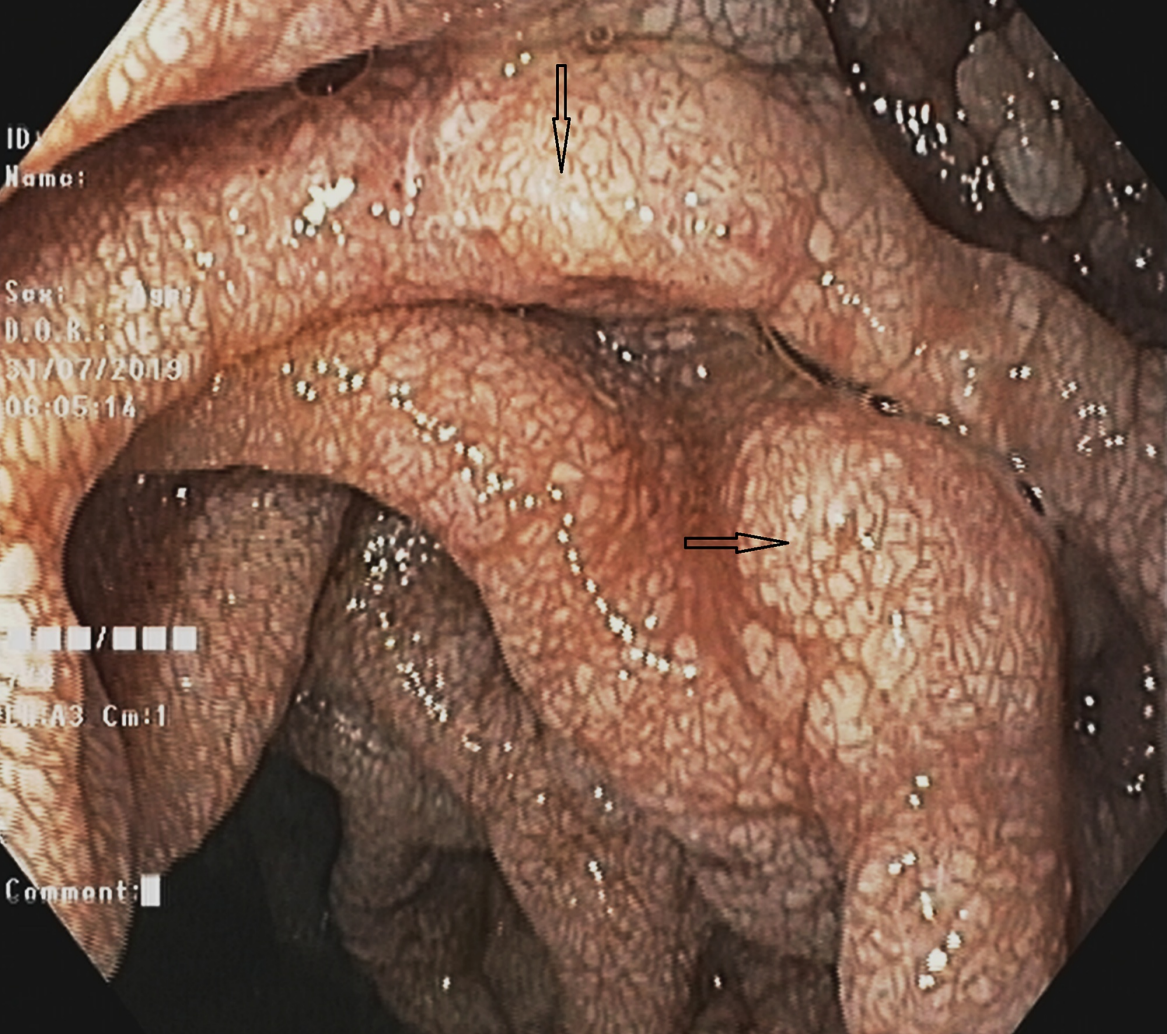
Upper gastrointestinal endoscopy showing circumferential polypoid mucosae covered with whitish swollen villi in the duodenum, suggesting intestinal lymphangiectasia.

**Figure 3:**
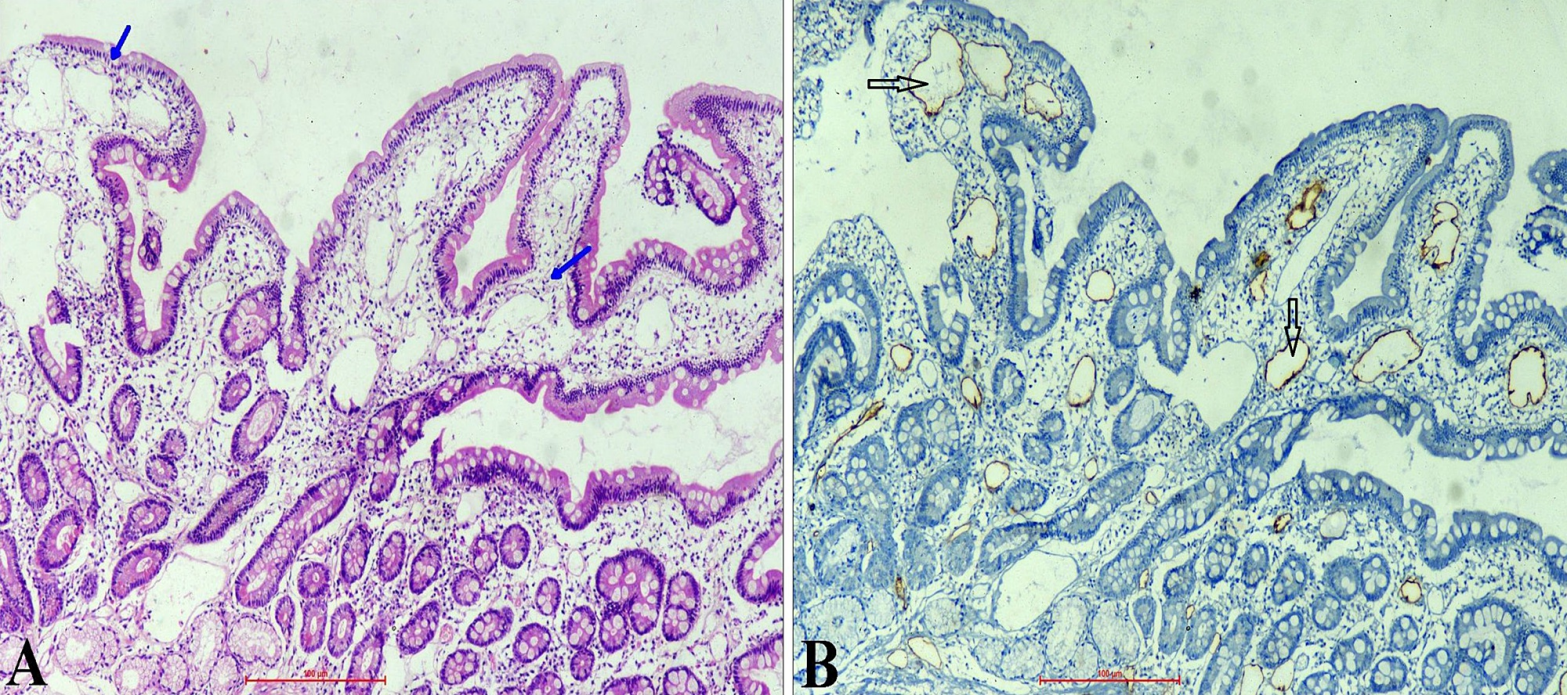
Histological examination (H&E: x10) of a duodenal biopsy specimen showing markedly dilated vessels in the lamina propria suggestive of intestinal lymphangiectasia (A). On immunohistochemistry (IHC: x10), the dilated vessels showing strong D2-40 positivity indicating dilated lymphatics (B).

A 59-year-old gentleman, known to have had cirrhosis, presented with progressive worsening of ascites and pedal edema for 15 days, and mild altered sensorium for three days. His ascites had deteriorated while being on diuretic therapy, and he had undergone repeated therapeutic paracentesis during recent times. On physical examination, he had generalized edema along with tense ascites and mild encephalopathy. In his blood tests, the important results were: hypoproteinemia (5.06 g/dL), hypoalbuminemia (2.24 g/dL), lymphocytopenia (280/µL) and raised international normalized ratio (INR 1.8). His kidney function tests, as well as serum electrolytes and liver enzymes, were within normal limits. His chest radiography revealed mild bilateral pleural effusion. Fluid analysis of ascites showed low protein (1.58 gm/dL), high serum ascites albumin gradient (SAAG: 1.41), lymphocyte predominant cells (100/µL) and triglyceride level (62 mg/dL). There was a significant amount of ascites on ultrasound and the liver appeared mildly shrunken with coarse echotexture. A computed tomography (CT) scan demonstrated thrombosed splenic and superior mesenteric veins with collaterals around (Figure 1). Transient elastography (FibroScan; Echosens, France), performed after large-volume paracentesis, revealed elevated liver stiffness (17.4 kPa). On upper gastrointestinal endoscopy, there were circumferential polypoid mucosae covered with whitish swollen villi in the duodenum, suggesting IL (Figure 2). In addition, there was evidence of severe portal hypertensive gastropathy, but no esophageal varices. Histological examination of a duodenal biopsy specimen showed markedly dilated vessels in the lamina propria, which on immunohistochemistry were strongly D2-40 positive suggestive of lymphatic vessels (Figures 3A, 3B). Based on the findings, a diagnosis of cirrhosis with PHT complicated by splenomesentric venous thrombosis and severe IL was made. Along with other regular treatment, he was initially treated with albumin infusion for three days. After an improvement in hepatic encephalopathy, diuretics were added and, once the diagnosis of IL was established, he was placed on a low-fat diet along with medium-chain triglycerides (MCT). Subsequently, his peripheral edema and ascites were steadily but substantially resolved over weeks.

## Discussion

In patients with early cirrhosis, lymphatic system helps to prevent the development of ascites by reabsorbing excess fluid in the hepatic and splanchnic areas. However, in advanced cirrhosis, this compensatory function is not adequate to avoid the development of ascites. Moreover, there is an impaired lymphatic transport function in patients with advanced cirrhosis [1,8]. The mechanism behind lymphatic dysfunction in cirrhosis is an area open for investigations. In a study on cirrhotic rats with ascites, Ribera et al found that an impaired lymphatic drainage in the splanchnic and peripheral regions was accompanied by increased production of nitric oxide by lymphatic endothelial cells [9]. Interestingly, when cirrhotic rats were treated with an inhibitor of nitric oxide synthase, a significant improvement of lymphatic drainage and reduction in ascitic volume were seen, suggesting a role of nitric oxide in the lymphatic dysfunction.

An increased intestinal lymphatic pressure due to PHT may lead to the development and then rupture of IL, causing loss of plasma proteins, lymphocytes and chylomicrons. Severe hypoalbuminemia combined with inadequate lymphatic drainage can greatly impair the fluid mobilization in patients with advanced cirrhosis. IL may thus contribute to refractory ascites in such patients. There was thrombosis of mesentricosplenic vein in our case, which may have induced added resistance to lymphaticovenous flow with subsequent aggravation of IL. In our case, IL was confirmed by histopathology and, thus, the mucosal change in the duodenum was not merely a passive congestion secondary to thrombosis of the mesentricosplenic vein. Although the cause of venous thrombosis could not be asserted, a decrease in portal blood flow leading to stasis and functional decrease in the amount of natural anticoagulants, including protein C, protein S and antithrombin III, contributes to venous thrombosis in cirrhosis [10]. In cirrhosis, evaluation of lymphatic dysfunction is often difficult. Clinically, the presence of lymphedema as demonstrated by the appearance of peau d’orange and the positive Stemmer sign may suggest lymphatic dysfunction. Chylous ascites occurs very rarely in cirrhosis due to the rupture of sub-serosal lymphatic vessels secondary to PHT. Chylous ascites appears milky white due to the high triglyceride content. Sometimes, pus can also give a milky appearance to ascitic fluid, known as pseudochylous ascites. In our case, however, ascitic fluid was neither chylous nor pseudochylous. Radiological approaches for evaluating the lymphatic system are still evolving and are often constrained by lack of standardization, technological challenges, inadequate resolution and low availability. And to date, there is no guideline for the use of different lymphangiographic methods in patients with cirrhosis. Endoscopically lymphangiectasia appears as whitish distended villi that can be confirmed on histopathological examination [11].

Dietary changes are currently the cornerstone of lymphangiectasia therapy. Because the dietary fat significantly influences the intestinal lymph flow, patients should be put on a low-fat diet [12]. MCT should be used for fat nutrition, as they are directly absorbed into the portal venous system without lacteal involvement. We believe that dietary intervention may have significantly contributed to the control of ascites in our patient, as the amount of ascites decreased gradually over weeks only after a low-fat diet and MCT had started. In addition, despite being on diuretic medication, he had experienced worsening of ascites, and he got albumin only for the first three days after he was admitted. The role of therapeutic albumin infusion in patients with lymphatic dysfunction is not very clear. The therapeutic value of albumin may be compromised by a high transcapillary escape rate and impaired albumin re-circulation from the intertitium to systemic circulation due to lymphatic dysfunction in patients with advanced cirrhosis. Octreotide has been found helpful in reducing the leakage of intestinal lymph [13]. Lastly, both liver transplantation and transjugular intrahepatic porto-systemic shunt have been found to improve IL and protein-losing enteropathy caused by PHT [14,15]. 

## Conclusions

IL is infrequently seen in patients with cirrhosis. Being associated with PHT, it may be worsened by the concomitant splenoportal axis thrombosis. Several complications, including refractory ascites, may result from a chronic intestinal loss of protein, lymphocytes and chylomicron via rupture of dilated lymphatics. For effective therapeutic intervention, timely diagnosis of this condition is important.
